# Supplementary material for: Efficacy of the Sentinox Spray in Reducing Viral Load in Mild COVID-19 and Its Virucidal Activity against Other Respiratory Viruses: Results of a Randomized Controlled Trial and an In Vitro Study
Source: Viruses. 2022 May 12;14(5):1033. doi: 10.3390/v14051033 (PMC9144724; doi:10.3390/v14051033)
Supplement: Supplementary file 1 [file viruses-14-01033-s001.zip › viruses-1706387-supplementary.pdf]

viruses

MDPI

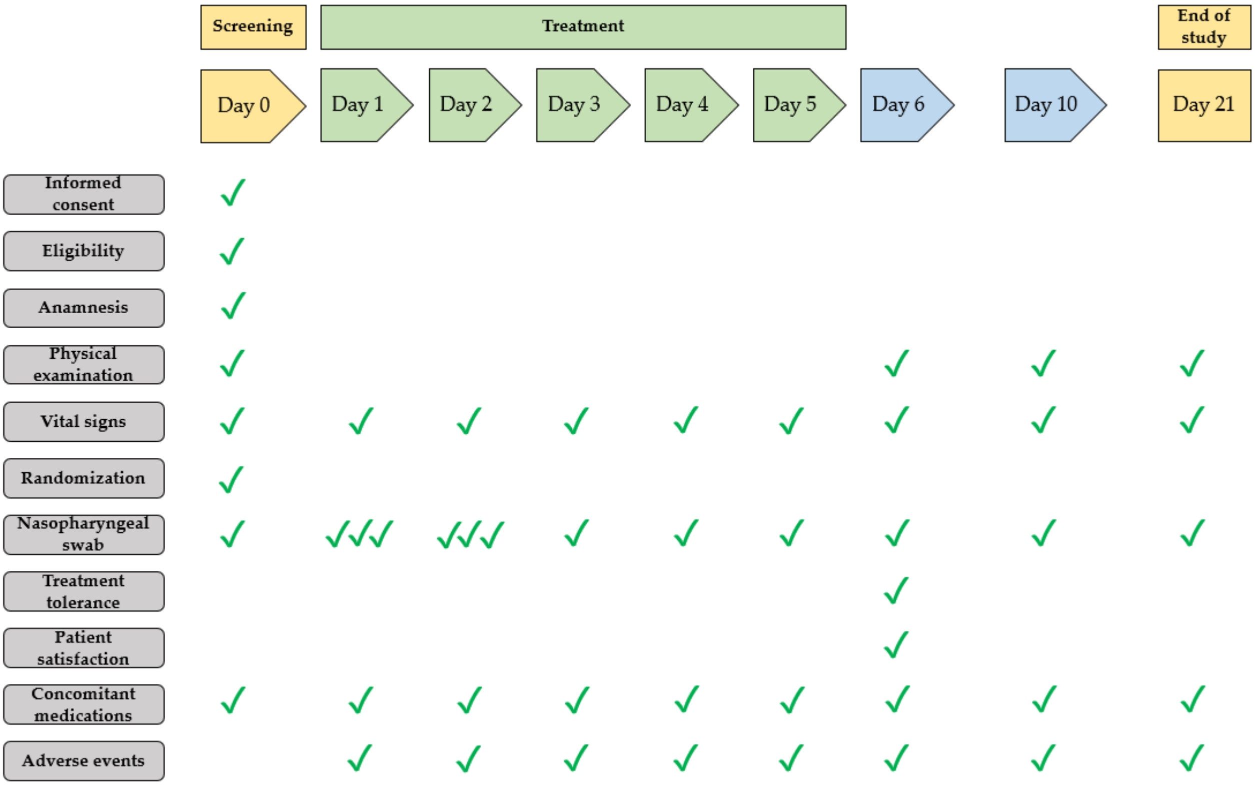

Figure S1. Overview of the study procedures.

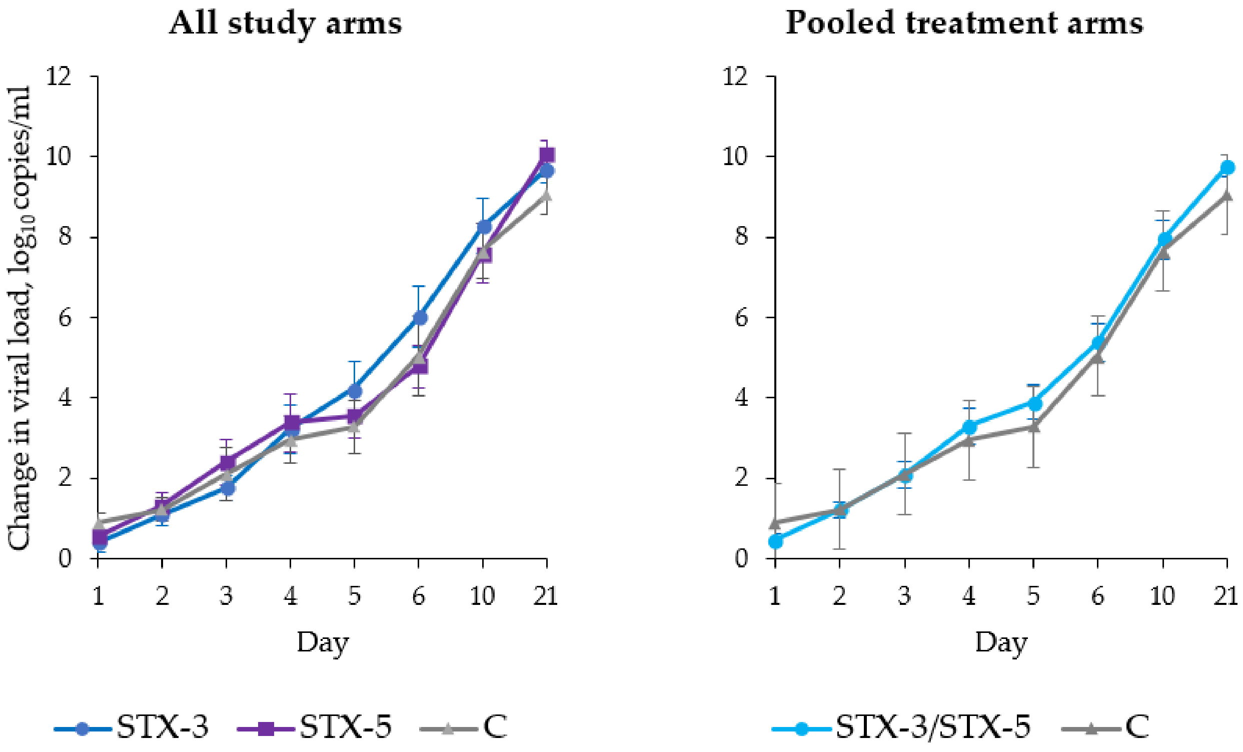

Figure S2. Absolute change in viral loads in the per-protocol population (n = 49), by study arm and day of follow-up (vertical bars represent standard errors).

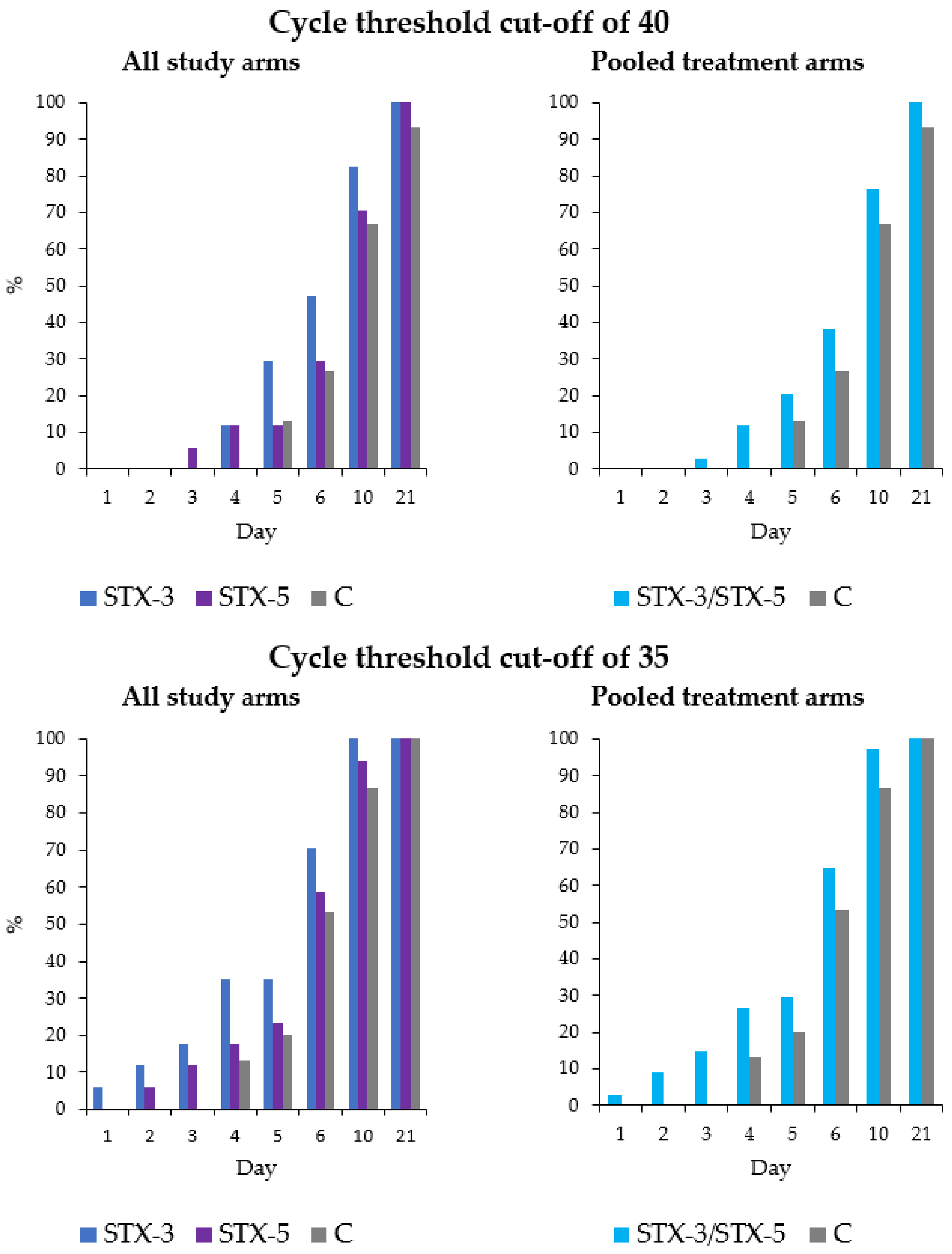

Figure S3. Proportion of negativized subjects in the per-protocol analysis, by negativization definition, study arm and day of follow-up.

Table S1. Test media used in the study, by virus.

Virus	Medium Components
Influenza virus A(H1N1)	Dulbecco's modified eagle medium (DMEM) supplemented with 2 μg/ml TPCK-trypsin, 100 units/ml penicillin, 10 μg/ml gentamicin, 2.5 μg/ml amphotericin B
Influenza virus B	Dulbecco's modified eagle medium (DMEM) supplemented with 2 μg/ml TPCK-trypsin, 100 units/ml penicillin, 10 μg/ml gentamicin, 2.5 μg/ml amphotericin B
RSV A	Dulbecco's modified eagle medium (DMEM) supplemented with 2% (v/v) heat-inactivated fetal bovine serum (FBS), 1.0 mM L-glutamine, 100 units/ml penicillin, 10 μg/ml gentamicin, 2.5 μg/ml amphotericin B
Rhinovirus	Minimal essential medium (MEM) supplemented with 10% (v/v) heat-inactivated fetal bovine serum (FBS), 100 units/ml penicillin, 10 μg/ml gentamicin, 2.5 μg/ml amphotericin B
Adenovirus 5	Minimal essential medium (MEM) supplemented with 5% (v/v) heat-inactivated fetal bovine serum (FBS), 100 units/ml penicillin, 10 μg/ml gentamicin, 2.5 μg/ml amphotericin B
Parainfluenza virus 3	Minimal essential medium (MEM) supplemented with 1% (v/v) heat-inactivated fetal bovine serum (FBS), 100 units/ml penicillin, 10 μg/ml gentamicin, 2.5 μg/ml amphotericin B
Coronavirus 229E	Minimal essential medium (MEM) supplemented with 2% (v/v) heat-inactivated fetal bovine serum (FBS), 100 units/ml penicillin, 10 μg/ml gentamicin, 2.5 μg/ml amphotericin B
